# Influence of *TGFBR2*, *TGFB3*, *DNMT1*, and *DNMT3A* Knockdowns on CTGF, TGFBR2, and DNMT3A in Neonatal and Adult Human Dermal Fibroblasts Cell Lines

**DOI:** 10.3390/cimb43010023

**Published:** 2021-06-03

**Authors:** Katarzyna Tomela, Justyna A. Karolak, Barbara Ginter-Matuszewska, Michal Kabza, Marzena Gajecka

**Affiliations:** 1 Chair and Department of Genetics and Pharmaceutical Microbiology, Poznan University of Medical Sciences, 60-781 Poznan, Poland; ktomela@gmail.com (K.T.); jkarolak@ump.edu.pl (J.A.K.); bgintermatuszewska@ump.edu.pl (B.G.-M.); mkabza@outlook.com (M.K.); 2 Institute of Human Genetics, Polish Academy of Sciences, 60-479 Poznan, Poland

**Keywords:** TGFB1, TGFB2, TGFB3, TGFBR2, CTGF, DNMT3A, DNMT1, human dermal fibroblasts

## Abstract

Dermal fibroblasts are responsible for the production of the extracellular matrix that undergoes significant changes during the skin aging process. These changes are partially controlled by the TGF-β signaling, which regulates tissue homeostasis dependently on several genes, including CTGF and DNA methyltransferases. To investigate the potential differences in the regulation of the TGF-β signaling and related molecular pathways at distinct developmental stages, we silenced the expression of *TGFB1*, *TGFB3*, *TGFBR2*, *CTGF*, *DNMT1*, and *DNMT3A* in the neonatal (HDF-N) and adult (HDF-A) human dermal fibroblasts using the RNAi method. Through Western blot, we analyzed the effects of the knockdowns of these genes on the level of the CTGF, TGFBR2, and DNMT3A proteins in both cell lines. In the *in vitro* assays, we observed that CTGF level was decreased after knockdown of *DNMT1* in HDF-N but not in HDF-A. Similarly, the level of DNMT3A was decreased only in HDF-N after silencing of *TGFBR2, TGFB3*, or *DNMT1*. TGFBR2 level was lower in HDF-N after knockdown of *TGFB3*, *DNMT1*, or *DNMT3A,* but it was higher in HDF-A after TGFB1 silencing. The reduction of TGFBR2 after silencing of *DNMT3A* and vice versa in neonatal cells only suggests the developmental stage-specific interactions between these two genes. However, additional studies are needed to explain the dependencies between analyzed proteins.

## 1. Introduction

Dermal fibroblasts are specialized cells within the dermis layer of the skin, responsible for the synthesis and remodeling of extracellular matrix (ECM) proteins. The function of skin dermis is modulated by its own cell-specific circadian clock, a system that determines gene expression fluctuation based on day and night rhythm [1]. In mammals, periodic transcriptomic changes are controlled by molecular network of clock genes, including *Bmal1* [2]. Throughout the lifespan, the processes regulating circadian system undergoe changes leading to decreased circadian function and skin aging [3].

During both wound healing and dermal aging, the ECM structure undergoes significant modifications [4,5]. Age-related alterations impair skin function and can result in improper wound healing [6]. It has been documented that the healing process is faster and more efficient at an early neonatal age compared to adults [7,8]. In addition, the transcriptome and protein profiles vary between dermal fibroblasts from newborns and older individuals, suggesting different mechanisms of gene activation depending on developmental stages [7,8].

The remodeling of dermal ECM is partially controlled by transforming growth factor β (TGF-β) signaling [5]. TGF-β superfamily of proteins is a large group of pleiotropic multifunctional cytokines containing more than 30 related growth factors [9], including the TGFB1, TGFB2, and TGFB3, which interact with TGF-β receptors, TGFBR1 and TGFBR2 [9]. TGF-β ligands initiate signaling by binding to their receptors, resulting in phosphorylation of SMAD proteins and the transduction of the SMAD complex into the nucleus [9]. This drives activation of a molecular cascade regulating downstream gene expression [9].

Interestingly, the TGF-β pathway’s activation induces connective tissue growth factor (CTGF), suggesting that CTGF also regulates functions of TGF-β and modulates gene expression. In particular, CTGF is a mediator of extracellular matrix (ECM) formation essential for the wound healing process, including skin repair after injury [10,11]. Since CTGF contains a domain that interacts with TGFB1, it could also enhance TGF-β signaling [12].

In addition to CTGF, TGF-β molecular pathway modulates the activity of DNA methyltransferases (DNMTs). The DNMT family of enzymes contains three isoforms, DNMT1, DNMT3A, and DNMT3B, responsible for the addition of methyl groups to DNA, resulting in enhancement or inhibition of a target gene expression [13]. The TGF-β signaling controls the earliest stages of human development and an adult tissue homeostasis by regulating cell proliferation, differentiation, migration, and apoptosis [14,15]. It is also involved in the epithelial–mesenchymal transition, a process in which epithelial cells undergo multiple biochemical changes and gain a mesenchymal cell phenotype [16].

Here, to better understand the regulation of genes involved in TGF-β signaling in human dermal fibroblasts (HDF) at different stages of their development, we used the RNAi-based method to silence *TGFB1, TGFB3, TGFBR2, CTGF*, and two DNA methyltransferases (*DNMT1* and *DNMT3A)* in the neonatal and adult HDF cell lines. Through in vitro experiments, we analyzed whether the knockdown of selected genes could affect the level of the CTGF, TGFBR2, and DNMT3A proteins and investigated if their levels vary in HDFs at different stages of their development.

## 2. Materials and Methods

### 2.1. Cell Culture

Human dermal fibroblast neonatal cell line (HDF-N) derived from neonatal foreskin (C-004-5C, Thermo Fisher Scientific, Carlsbad, CA, USA), and human dermal fibroblast adult cell line (HDF-A) isolated from adult skin (C-013-5C, Thermo Fisher Scientific, Carlsbad, CA, USA) were cultured in a humidified atmosphere under the standard conditions (37 °C, 5% CO_2_) in Medium 106 (Thermo Fisher Scientific, Carlsbad, CA, USA) supplemented with Low Serum Growth Supplement (Thermo Fisher Scientific, Carlsbad, CA, USA), according to manufacturer’s instruction. The number of passages of cells that were used in analyses were 11 and 14 for (HDF-N) and 14 (HDF-A,) respectively.

### 2.2. Transfection

Cells were grown to 80%–90% confluence, and then were harvested using Trypsin/EDTA Solution (Thermo Fisher Scientific, Carlsbad, CA, USA), re-plated, and cultured for 24 h. On the day of transfection, cells were harvested, washed twice in DPBS (Thermo Fisher Scientific, Carlsbad, CA, USA), and resuspended in Buffer R (Thermo Fisher Scientific, Carlsbad, CA, USA). Electroporation was performed using the Neon Transfection System (Thermo Fisher Scientific, Carlsbad, CA, USA) with a time constant protocol at 1400 V for 30 ms per 1 million of HDF-A cells and 1700 V for 20 ms per 0.75 million of HDF-N cells per transfection. Each cell line was transfected with 30 nM of one of six small interfering RNAs (siRNAs), targeting transcripts of *CTGF*, *TGFBR2*, *TGFB1*, *TGFB3*, *DNMT1*, and *DNMT3A* (Santa Cruz Biotechnology, Dallas, TX, USA). A randomized siRNA (Santa Cruz Biotechnology, Dallas, TX, USA) not targeting any specific gene product was used as an internal control. Detailed information about the applied siRNAs is shown in Supplementary Materials Table S1.

### 2.3. Western Blot

Forty-eight hours after transfection, cells were washed twice in DPBS and lysed in ice-cold RIPA buffer (150 mM sodium chloride, 1% Triton X-100, 0.5% sodium deoxycholate, 0.1% SDS, 50 mM Tris pH 8.0) with a protease inhibitors cocktail (BioShop, Burlington, ON, Canada). Samples were denatured with a 4× Laemmli buffer (4% SDS, 20% glycerol, 0.004% bromophenol blue, 0.125M Tris HCl, 10% 2-mercaptoethanol), and 55 µg of total protein per lane was loaded onto 8% or 12% SDS polyacrylamide gels. After electrophoresis, separated proteins were transferred onto nitrocellulose membranes (Bio-Rad, Hercules, CA, USA) and incubated overnight with one of the following primary mouse antibodies: anti-Ctgf, anti-Tgfbr2, and anti-Dnmt3a (Santa Cruz Biotechnology, Dallas, TX, USA) at 4 °C. Blots were washed three times in a 1× TBST and incubated with horseradish peroxidase (HRP) conjugated secondary antibody (anti-mouse IgG, Santa Cruz Biotechnology, Dallas, TX, USA). Proteins were enzymatically detected by Clarity Western ECL Substrate system (Bio-Rad, Hercules, CA, USA) and semi-quantitative measurement of protein level was performed in Image Lab 5.1 software (Bio-Rad, Hercules, CA, USA). Then, the membranes were re-incubated with primary rabbit antibody anti-β-Actin (Merck, Darmstadt, Germany) and HRP-conjugated secondary anti-rabbit IgG (Merck, Darmstadt, Germany) as described above, to measure the level of the reference protein. Detailed information about the applied antibody dilutions is presented in Table S2.

### 2.4. Protein Specificity Test

To ensure that analyzed proteins were specific, a protein specificity test was performed. The protein level of silenced genes in HDF-N and HDF-A cells was assessed with antibodies anti-Ctgf, anti-Tgfbr2, anti-Dnmt3a, as recommended by the manufacturer. To verify the specific protein band for each antibody, a part of the membrane with an additional protein sample was incubated only with the secondary antibodies. Levels of protein products were reduced in samples with lysates from cells transfected with siRNA related to a particular protein and did not constitute unspecific product(-s) detected by the secondary antibody itself.

### 2.5. Endogenous In Vitro Protein Level

To compare endogenous protein level in untreated HDF-N and HDF-A cell lines, 55 µg of protein from each culture were separated on SDS polyacrylamide gel, transferred to nitrocellulose membrane and immunostained as described above. The CTGF, TGFBR2, and DNMT3A protein levels in the HDF-N cell line were considered as 100% in the calculation of protein levels for the HDF-A cell line. Since the level of the actin-β protein was used as a reference, each membrane was re-incubated with primary rabbit anti-β-Actin antibody and HRP-conjugated secondary anti-rabbit IgG (Merck, Darmstadt, Germany), as described above.

### 2.6. Statistical Analysis

All experiments except one were performed at least three times. Analysis of the endogenous level of DNMT3A protein in HDF-N and HDF-A cells was performed once, without duplicates, due to the lack of reagent (discontinuation of antibody production by the manufacturer). Results were analyzed by normalizing the value of the optical density of the individual proteins to actin-β level. After the knockdown, the protein levels were calculated by comparison of the expression to the specific endogenous protein levels (with values of 100%) in cells transfected with the control siRNA. The statistical significance of the differential protein-level expression after RNAi mediated silencing of the target genes was evaluated using Student’s *t*-test. Differences with *p*-values lower than 0.05 after Benjamini-Hochberg correction were considered as statistically significant.

## 3. Results

### 3.1. Protein Specificity Test

Double verification of the specificity of analyzed proteins was performed. Firstly, we confirmed that protein bands in the silenced cell lysates had reduced density signals comparing to protein bands from the untreated cells. The CTGF protein level was reduced by 93% and 75% in the neonatal (HDF-N) and adult (HDF-A) human dermal fibroblasts cell lines, respectively. The TGFBR2 protein level was decreased by 98% in HDF-N and 93% in HDF-A cells. The DNMT3A protein level was also reduced in both cell lines by 95% (HDF-N) and 88% (HDF-A). Downregulation of CTGF, TGFBR2, and DNMT3A protein levels was statistically significant (*p* < 0.05).

**Figure 1:**
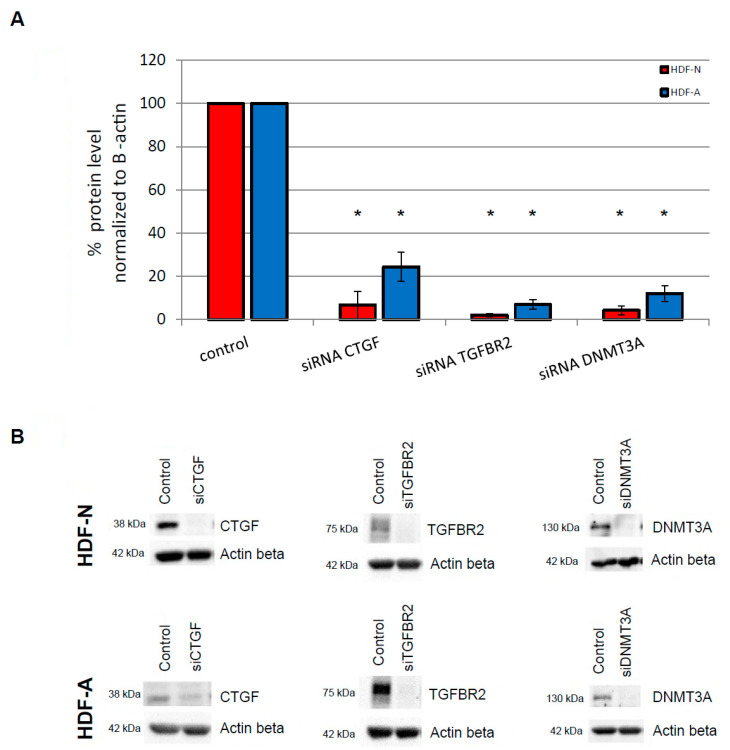
Verification of specificity of siRNA on CTGF, TGFBR2, and DNMT3A proteins in neonatal (HDF-N) and adult (HDF-A) human dermal fibroblasts. Cells treated with control siRNA were considered as an experimental control (with a value of 100%) in comparison with the protein levels in cells treated with various specific siRNAs. Protein levels were normalized to Actin β. (**A**) The protein levels of CTGF, TGFBR2, and DNMT3A after silencing with specific siRNAs are significantly reduced in HDF-N and HDF-A cells (* *p*-value < 0.05). Error bars represent standard error of the mean. (**B**) Representative immunoblots show the specificity of analyzed proteins: significantly reduced protein level in cells treated with various specific siRNAs in the evaluated cell lines.

Moreover, the specificity of protein bands was verified by the assessment of the unspecific protein products in the membrane fragments treated with the secondary antibody alone. We did not observe any unspecific products in the size of indicated proteins (Figure 1).

### 3.2. Endogenous In Vitro Levels of Proteins Associated with the TGF-β Pathway and DNA Methyltransferases

**Figure 2:**
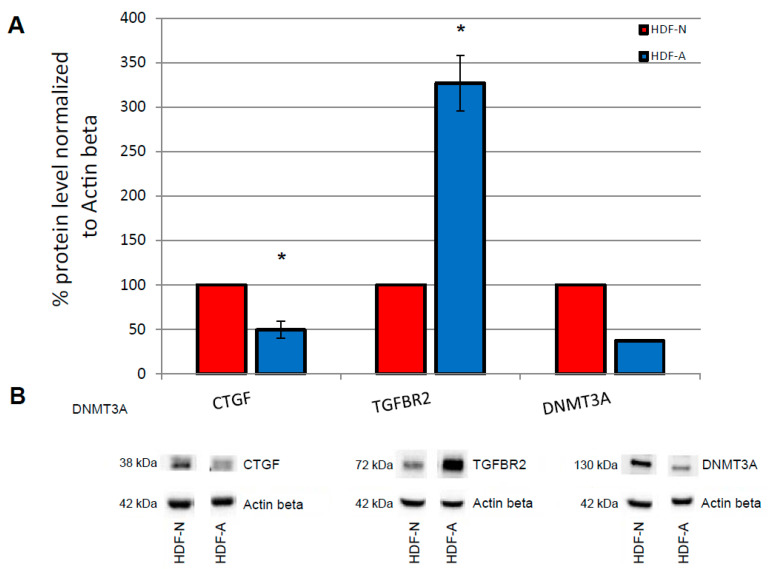
The endogenous in vitro level of CTGF, TGFBR2 and DNMT3A proteins in neonatal (HDF-N) and adult (HDF-A) human dermal fibroblasts. (**A**) The endogenous protein level for all analyzed proteins was normalized to Actin β. The protein level in HDF-N cells was considered as 100%. Endogenous CTGF protein level is significantly lower in HDF-A cells compared to HDF-N, contrary to endogenous TGFBR2 protein level which is significantly higher in HDF-A compared to HDF-N cells (* *p*-value < 0.05). Error bars represent standard error of the mean. All but one (DNMT3A) experiment was performed in triplicate. (**B**) The immunoblots show differences in endogenous protein levels between analysed cell lines.

Endogenous CTGF protein level was 52% lower in the HDF-A cells compared to HDF-N cells (*p* = 0.017). Similarly, endogenous DNMT3A protein level was 63% lower in the HDF-A cells than in the HDF-N cells. In contrast, endogenous TGFBR2 protein level was 227% higher in the HDF-A cells compared to the HDF-N cells (*p* = 0.023) (Figure 2).

### 3.3. The In Vitro Effect of Analyzed Genes on the CTGF, TGFBR2, and DNMT3A Protein Levels

**Figure 3:**
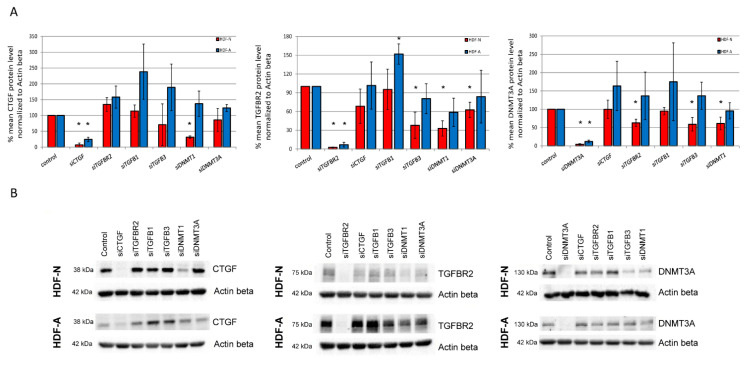
Western blot results (**A**) Quantified levels of the CTGF, TGFBR2 and DNMT3A proteins after siRNA-mediated silencing of *CTGF*, *TGFBR2*, *TGFB1*, *TGFB3*, *DNMT1*, and *DNMT3A*. The protein band densities were assessed in ImageLab software and % mean protein level was calculated in comparison to a control protein level. All protein levels were normalized to Actin β. The protein level in cells treated with control siRNA was considered as a control (100%). Error bars represent standard error of the mean, * *p*-value < 0.05. Data are representative of at least three independent experiments. (**B**) The immunoblots of CTGF, TGFBR2 and DNMT3A protein levels in neonatal (HDF-N) and adult (HDF-A) human dermal fibroblast cell lines in response to an in vitro treatment with *CTGF* siRNA, *TGFBR2* siRNA, *TGFB1* siRNA, *TGFB3* siRNA, *DNMT1* siRNA, *DNMT3A* siRNA, and control siRNA.

The CTGF protein level was reduced by 68% in HDF-N cells after the silencing of *DNMT1*. No effect of *DNMT1* on the CTGF protein amount was observed in HDF-A cell line. We did not observe any change in CTGF level in both examined cell lines after the silencing of *TGFBR2*, *TGFB1*, *TGFB3*, and *DNMT3A.* A 52% increase in the TGFBR2 protein level in HDF-A cells was detected after siRNA knockdown of *TGFB1*. No similar effect of *TGFB1* siRNA on TGFBR2 was observed in the HDF-N cell line. The use of siRNA targeting the *TGFB3*, *DNTM1*, and *DNMT3A* transcripts resulted in a 62%, 67%, and 37% reduction of the TGFBR2 protein level in HDF-N cells, respectively, while no changes in TGFBR2 amount were identified in HDF-A cell line after the treatment with the same siRNAs. The TGFBR2 protein level also remained unchanged after using the *CTGF* siRNA in both neonatal and adult cells. The DNMT3A protein level was decreased by 37% in HDF-N cells after the silencing of *TGFBR2*, but no change was observed in HDF-A cells. The siRNA-mediated knockdown of *TGFB3* and *DNMT1* resulted in a 41% and 39% reduction of the DNMT3A protein level in HDF-N cells. No effect of *TGFB3* or *DNMT1* silencing on the DNMT3A protein amount was observed in HDF-A cells. The level of DNMT3A also remained unchanged in both cell lines after the treatment with *CTGF* and *TGFB1* siRNAs. All findings concerning the protein levels of particular proteins were statistically significant (*p* < 0.05; calculated *p*-values are presented in Table 1). The results are summarized in Figure 3 and Table 1.

## 4. Discussion

The TGF-β signaling is an essential molecular transduction cascade regulating organogenesis and tissue homeostasis. Depending on the affected cells or tissue types, its disruption can lead to various diseases [17,18,19]. In carcinogenesis, deregulation of TGF-β is implicated in both inhibiting cell cycle progression (tumor-suppressive effects) and increasing cell metastasis (tumor-promoting effects) [17]. While the alterations of the TGF-β signaling in corneal cells result in corneal dystrophies and keratoconus [20,21,22], disturbances of the TGF-β pathway in the lung may contribute to tissue fibrosis [23].

TGF-β also regulates the composition of skin ECM and the expression pattern of genes involved in the TGF-β signaling undergoes dynamic changes in the dermal fibroblasts at different developmental stages [5]. Here, to investigate the potential differences in the regulation of the TGF-β pathway, we used a functional post-transcriptomic approach and assessed the effect of the in vitro silencing of selected genes on the CTGF, TGFBR2, and DNMT3A protein levels in neonatal and adult human dermal fibroblast cell lines, HDF-N and HDF-A.

Our investigation showed that the endogenous level of the CTGF protein has been lower in HDF-A compared to HDF-N. These results are in agreement with previous findings showing decreased CTGF level in aged human skin in vivo and adult dermal fibroblasts [24,25,26]. CTGF is induced by TGF-β and seems to function as a downstream mediator of TGF-β signaling in dermal fibroblasts [24]. While knockdown of *TGFB1* or *TGFB3* reduced CTGF level in skin fibroblasts, the 24-hour stimulation of these cells by TGF-β treatment resulted in upregulation of CTGF [25,27]. Interestingly, in our study, no evidence of changed CTGF protein level after silencing of TGF-β members (either *TGFB1*, *TGFB3*, or *TGFBR2*) in neonatal or adult HDF cells was found. It was shown that the reduction of CTGF level in skin fibroblasts after knockdown of each TGF-β isoform separately is lower than after knockdown of TGF-β1, TGF-β2, and TGF-β3 in combination [25]. In our study, each knockdown was performed separately and that could partially explain the lack of changes in the CTGF expression after siRNA-mediated silencing of *TGFB1* or *TGFB3.* In addition, basal expression of CTGF could also be regulated through TGF-β independent pathways [28], which could mask the effect of *TGFB1*, *TGFB3*, or *TGFBR2* knockdowns.

In the TGF-β pathway, both TGFB1 and TGFB3 can bind to TGFBR2 and activate the signaling cascade. TGFB1 has been recognized as a ligand stimulating a profibrotic response following injury, whereas TGFB3 is known for its antifibrotic effects [29]. Dermal fibroblasts from neonates have higher TGFB3 and lower TGFBR2 mRNA levels than fibroblasts obtained from adults, suggesting that an antifibrotic mechanism is favored in younger cells [26]. Indeed, it was shown that decreased expression of TGFBR2 in neonatal dermal cells is associated with reduced scarring in neonates [26]. The cause of various endogenous levels of TGFBR2 in neonatal and adult HDF observed in this and previous studies [26,30] is unknown. Here, we showed that the silencing of *TGFB1* or *TGFB3* results in the oppositely altered protein level of TGFBR2 in dermal fibroblasts. After using siRNA targeting *TGFB1*, the TGFBR2 protein level was significantly increased in HDF-A but remained unchanged in HDF-N cells. In contrast, the amount of TGFBR2 was significantly lower in HDF-N cells treated with *TGFB3* siRNA, while no changes in TGFBR2 level were observed in HDF-A cells silenced with the same siRNA. These results imply that TGFBR2 expression level could depend on the different expression of the TGF-β members, TGFB3 in neonatal, and TGFB1 in adult dermal fibroblasts.

The earlier studies have also shown that the TGF-β expression pattern could depend on the methylation driving by three DNA methyltransferases. DNMT1 is a maintenance-type methyltransferase responsible for copying pre-existing DNA methylation patterns during DNA replication, while DNMT3A and DNMT3B are essential for introducing de novo methylation [31].

Our studies revealed that the level of the TGFBR2 in neonatal cells could be dependent on the level of DNMT3A. We observed decreased level of TGFBR2 in HDF-N after the RNAi-mediated silencing of *DNMT3A*, suggesting that this gene might affect in vitro TGFBR2 expression and influence the TGF-β signaling. Interestingly, RNAi-mediated silencing of *TGFBR2* also reduced the protein level of DNMT3A in neonatal HDF, pointing to two-sided interactions between TGFBR2 and DNMT3A in younger dermal cells. We have also observed decreased expression of TGFBR2, CTGF, and DNMT3A after *DNMT1* silencing in HDF, implying that the DNMT1 level could influence the level of these proteins. Reduced expression of DNMT3A after knockdown of *TGFB3* has also been observed. Again, this effect was revealed in neonatal cells only.

It was reported that TGFB1, one of the relevant ligands responsible for the initiation of the TGF-β signaling, has affected transcriptome by decreasing the levels of DNMT1 and DNMT3A in cardiac fibroblasts [32]. An opposite effect was observed in lung fibroblasts in which TGFB1 enhances a global DNMT1 and DNMT3A activity [33]. Similar epigenetic alterations also play an essential role in activating skin fibroblasts in systemic sclerosis [34]. While the cooperation between DNMT and TGFB1 was studied earlier, according to our knowledge, the interrelationship of other TGF-β members with DNMT genes in HDF has not been evaluated before. Our results indicated differences in maintaining the TGF-β pathway in neonatal HDF cells compared to the adult ones and showed possible interactions between DNMT and TGF-β signaling. siRNAs are now routinely used in functional analyses in mammalian cells. While siRNAs suppress mRNA levels for specific genes, several studies have demonstrated that siRNAs also have off-target effects [35,36]. Here, in addition to the target gene, we studied a few related genes to assess their function. However, we cannot exclude that other transcripts could also influence the observed interrelationship between analyzed proteins. Further research should be performed to add information to the obtained data.

## 5. Conclusions

In conclusion, we showed that the in vitro level of TGFBR2, CTGF, and DNMT3A in HDF is developmental stage-specific and could depend on the protein levels of other members of the TGF-β signaling pathway and related molecular cues. These findings allowed us to better understand the age-related changes in human dermal fibroblasts associated with TGF-β. However, since our research was limited to chosen members of the TGF-β pathway, further functional studies are needed to provide mechanistic insight into mutual relations between analyzed genes in dermal fibroblasts. In addition, because the changes in protein levels were observed in vitro, in vivo studies are needed to fully recognize the impact of detected differences in protein profiles in neonatal and adult HDF cells.

## Figures and Tables

**Table 1:** Effects of the in vitro siRNA mediated knockdowns of *CTGF*, *TGFBR2*, *TGFB1*, *TGFB3*, *DNMT1*, and *DNMT3A* on CTGF, TGFBR2, and DNMT3A protein levels in neonatal (HDF-N), and adult (HDF-A) cell lines. Statistically significant increased and decreased protein levels are indicated by symbols ↑, and ↓, respectively; N/E, no effect.

Analyzed Protein		Specific Gene Targeted siRNA					
		si*CTGF*	si*TGFBR2*	si*TGFB1*	si*TGFB3*	si*DNMT1*	si*DNMT3A*
CTGF	HDF-N	↓ *p* = 0.001	N/E	N/E	N/E	↓ *p* = 0.001	N/E
	HDF-A	↓ *p* = 0.003	N/E	N/E	N/E	N/E	N/E
TGFBR2	HDF-N	N/E	↓ *p* < 0.001	N/E	↓ *p* = 0.053	↓ *p* = 0.015	↓ *p* = 0.053
	HDF-A	N/E	↓ *p* < 0.001	↑ *p* = 0.050	N/E	N/E	N/E
DNMT3A	HDF-N	N/E	↓ *p* = 0.011	N/E	↓ *p* = 0.050	↓ *p* = 0.050	↓ *p* < 0.001
	HDF-A	N/E	N/E	N/E	N/E	N/E	↓ *p* = 0.007

## Data Availability

The data presented in this study are available on request from the corresponding author.

## Supplementary Materials

The following are available online at https://www.mdpi.com/article/10.3390/cimb43010023/s1, Table S1: Sequences of siRNAs applied in the transfection of human dermal fibroblasts, Table S2: Antibodies used for the western blot assays.
