# Supplementary material for: Influence of TGFBR2, TGFB3, DNMT1, and DNMT3A Knockdowns on CTGF, TGFBR2, and DNMT3A in Neonatal and Adult Human Dermal Fibroblasts Cell Lines
Source: Curr Issues Mol Biol. 2021 Jun 3;43(1):276–85. doi: 10.3390/cimb43010023 (PMC8928948; doi:10.3390/cimb43010023)
Supplement: Supplementary file 1 [file cimb-43-00023-s001.zip › Table S1_jun01.2021.pdf]

Table S1. Sequences of siRNAs applied in the transfection of human dermal fibroblasts

siRNA	Catalog number	Catalog number of individual siRNA duplex	Sequence in 5′ → 3′ orientation
CTGF siRNA	sc-39329	sc-39329A	Sense: CCAAGCCUAUCAAGUUUGAtt
			Antisense: UCAAACUUGAUAGGCUUGGtt
		sc-39329B	Sense: GAAGACAUACCGAGCUAAAtt
			Antisense: UUUAGCUCGGUAUGUCUUCtt
		sc-39329C	Sense: CACCAUAGGUAGAAUGUAAtt
			Antisense: UUACAUUCUACCUAUGGUGtt
TGF-ßRII siRNA	sc-36657	sc-36657A	Sense: CUGUAUGGAGAAAGAAUGAtt
			Antisense: UCAUUCUUUCUCCAUACAGtt
		sc-36657B	Sense: CUCCAAAGUGCAUUAUGAAtt
			Antisense: UUCAUAAUGCACUUUGGAGtt
		sc-36657C	Sense: CCAACAACAUCAACCACAAtt
			Antisense: UUGUGGUUGAUGUUGUUGGtt
TGF-ß1 siRNA	sc-270322	sc-270322A	Sense: CUGCAAGACUAUCGACAUGtt
			Antisense: CAUGUCGAUAGUCUUGCAGtt
		sc-270322B	Sense: GCAACAAUUCCUGGCGAUAtt
			Antisense: UAUCGCCAGGAAUUGUUGCtt
		sc-270322C	Sense: GACACCAACUAUUGCUUCAtt
			Antisense: UGAAGCAAUAGUUGGUGUCtt
TGF-ß3 siRNA	sc-39804	sc-39804A	Sense: GGAAUACUAUGCCAAAGAAtt
			Antisense: UUCUUUGGCAUAGUAUUCCtt
		sc-39804B	Sense: GGAAUUACCUCCAAGGUUUtt
			Antisense: AAACCUUGGAGGUAAUUCCtt
		sc-39804C	Sense: GAAUCACGGUGGUAAAGAAtt
			Antisense: UUCUUUACCACCGUGAUUCtt
DNMT1 siRNA	sc-156049	sc-156049A	Sense: GAAGAGACGUAGAGUUACAtt
			Antisense: UGUAACUCUACGUCUCUUCtt
		sc-156049B	Sense: CCUUCACGUUCAACAUCAAtt
			Antisense: UUGAUGUUGAACGUGAAGGtt
		sc-156049C	Sense: GUGUGAGGUUCGCUUAUCAtt
			Antisense: UGAUAAGCGAACCUCACACtt
DNMT3A siRNA	sc-37757	sc-37757A	Sense: CUCAGAGCUAUUACCCAAUtt
			Antisense: AUUGGGUAAUAGCUCUGAGtt
		sc-37757B	Sense: GAAGAAUCCCUACAAAGAAtt
			Antisense: UUCUUUGUAGGGAUUCUUCtt
		sc-37757C	Sense: CAUCCACUGUGAAUGAUAAtt
			Antisense: UUAUCAUUCACAGUGGAUGtt
Control siRNA-A	sc-37007	Confidential manufacturer' information	
