# Supplementary material for: Influence of TGFBR2, TGFB3, DNMT1, and DNMT3A Knockdowns on CTGF, TGFBR2, and DNMT3A in Neonatal and Adult Human Dermal Fibroblasts Cell Lines
Source: Curr Issues Mol Biol. 2021 Jun 3;43(1):276–85. doi: 10.3390/cimb43010023 (PMC8928948; doi:10.3390/cimb43010023)
Supplement: Supplementary file 1 [file cimb-43-00023-s001.zip › Table S2_jun01.2021.pdf]

Table S2. Antibodies used for the western blot assays

Primary antibodies			
Antibody	Catalog number	Concentration	Producer
anti-CTGF (E-5)	sc-365970	1/100	Santa Cruz Biotechnology, Dallas, TX
anti-TGF-ßRII (E-6)	sc-17792	1/200	Santa Cruz Biotechnology
anti-Dnmt3a (A-10)	sc-373905	1/100	Santa Cruz Biotechnology
anti-ß-Actin	A2066	1/10000	Sigma-Aldrich, St. Louis, MO
Secondary antibodies			
Antibody	Catalog number	Concentration	Producer
anti-mouse IgG-HRP	sc-2005	1/10000	Santa Cruz Biotechnology
anti-rabbit IgG-HRP	A6154	1/10000	Sigma-Aldrich
